# Bimetallic Aluminum‐ and Niobium‐Doped MCM‐41 for Efficient Conversion of Biomass‐Derived 2‐Methyltetrahydrofuran to Pentadienes

**DOI:** 10.1002/anie.202212164

**Published:** 2022-11-17

**Authors:** Mengtian Fan, Shaojun Xu, Bing An, Alena M. Sheveleva, Alexander Betts, Joseph Hurd, Zhaodong Zhu, Meng He, Dinu Iuga, Longfei Lin, Xinchen Kang, Christopher M. A. Parlett, Floriana Tuna, Eric J. L. McInnes, Luke L. Keenan, Daniel Lee, Martin P. Attfield, Sihai Yang

**Affiliations:** ^1^ Department of Chemistry University of Manchester Manchester M13 9PL UK; ^2^ Department of Chemical Engineering University of Manchester Manchester M13 9PL UK; ^3^ Department of Physics University of Warwick Coventry CV4 7AL UK; ^4^ Beijing National Laboratory for Molecular Sciences Key Laboratory of Colloid and Interface and Thermodynamics Institute of Chemistry Chinese Academy of Sciences Beijing 100190 China; ^5^ Diamond of Light Source, Harwell Science and Innovation Campus Oxfordshire OX11 0DE UK; ^6^ University of Manchester at Harwell, Harwell Science and Innovation Campus Oxfordshire OX11 0DE UK

**Keywords:** 2-Methyltetrahydrofuran, Cleavage of C−O Bond, Niobium Sites, Pentadienes

## Abstract

The production of conjugated C4–C5 dienes from biomass can enable the sustainable synthesis of many important polymers and liquid fuels. Here, we report the first example of bimetallic (Nb, Al)‐atomically doped mesoporous silica, denoted as AlNb‐MCM‐41, which affords quantitative conversion of 2‐methyltetrahydrofuran (2‐MTHF) to pentadienes with a high selectivity of 91 %. The incorporation of Al^III^ and Nb^V^ sites into the framework of AlNb‐MCM‐41 has effectively tuned the nature and distribution of Lewis and Brønsted acid sites within the structure. Operando X‐ray absorption, diffuse reflectance infrared and solid‐state NMR spectroscopy collectively reveal the molecular mechanism of the conversion of adsorbed 2‐MTHF over AlNb‐MCM‐41. Specifically, the atomically‐dispersed Nb^V^ sites play an important role in binding 2‐MTHF to drive the conversion. Overall, this study highlights the potential of hetero‐atomic mesoporous solids for the manufacture of renewable materials.

## Graphical abstract

The highly selective conversion of biomass‐derived 2‐methyltetrahydrofuran (2‐MTHF) into pentadienes has been achieved over an aluminum and niobium bimetallic atomically doped MCM‐41. The Nb^V^ sites enhance the catalytic performance by binding 2‐MTHF.

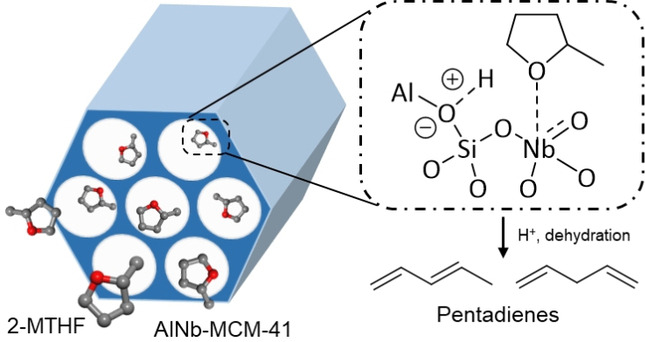

## Introduction

The global Net Zero Target promotes the utilisation of renewable resources, such as lignocellulosic biomass, to produce liquid fuels and chemical feestocks.[^1^, ^2^, ^3^] Pentadienes, particularly 1, 3‐pentadienes, serve as key building blocks for the synthesis of various adhesives, plastics, and resins as well as a range of fine chemicals through Diels–Alder reactions, Heck‐type reactions and hydroformylation.[^4^, ^5^] Currently, pentadienes are mainly obtained from the steam cracking of naphtha produced in the petroleum industry. Powerful drivers therefore exist for their production from biomass materials. 2‐Methyltetrahydrofuran (2‐MTHF) can be obtained readily from lignocellulose‐derived furfural in high yields and is used widely as a biofuel component and green solvent.[^6^, ^7^, ^8^] Emerging developments are reported for the conversion of 2‐MTHF to pentadienes via concerted ring‐opening, hydride transfer and dehydration reactions over various heterogeneous catalysts incorporating acid sites.^[9]^ For example, a V−Ti−P mixed oxide catalyst achieved a yield of 65.1 % for pentadienes at 350 °C and 1 atm.^[10]^ Amorphous SiO_2_/Al_2_O_3_ has shown a yield of 68 % for pentadienes in the initial ten hours of the reaction with full conversion of 2‐MTHF at 350 °C under 1 atm with pentenols, pentanal, pentenals, and butenes as well as C2–C3 alkenes detected as by‐products.^[5]^ Aluminium‐ and boron‐doped zeolites (MWW, MFI and BEA‐frameworks) have also been studied and the boron sites are shown to promote the selectivity of pentadienes.^[9]^ Recently, a phosphonate‐modified metal–organic framework, P‐UiO66‐PSM‐AW, has shown high selectivity to pentadienes (≈90 %) at 25 % conversion of 2‐MTHF at 280 °C.^[11]^ The strength of acidity of the catalysts plays an important role in the selectivity of dienes. To avoid further oligomerisation of the produced dienes on strong acid sites, catalysts with weakly acidic nature are preferred for this reaction. Furthermore, microporous catalysts often suffer from deactivation due to the formation of cokes that block the active sites within the micropores.

MCM‐41 is a mesoporous silica‐based material that has been used as catalyst or catalyst support for a wide range of reactions.^[12]^ Emerging niobium‐based catalysts exhibit excellent activities for the hydrodeoxygenation of biomass and related substrates under mild conditions owing to the strong capability of pentavalent Nb^V^ sites for the efficient activation of C−O bonds.[^13^, ^14^, ^15^, ^16^, ^17^] Nb_2_O_5_ and NbOPO_4_ have been tested for the conversion of 2‐MTHF to pentadienes but both suffer from rapid and complete deactivation owing to coke deposition on the surface, thus blocking the active sites.^[5]^ Here, we report the synthesis of the first example of bimetallic (Nb,Al)‐atomically doped mesoporous silica (denoted as AlNb‐MCM‐41). The optimisation of the nature and distribution of acid sites within MCM‐41 has been achieved by fine‐tuning the ratio of Nb^V^ and Al^III^ sites in the framework. AlNb‐MCM‐41(35/1/0.9) (the number in parenthesis represents the atomic ratio of Si/Al/Nb) enables the quantitative conversion of 2‐MTHF with a high selectivity of 91 % to pentadienes at 275 °C and 1 atm. Owing to the atomically‐dispersed Nb^V^ sites within the mesoporous matrix, AlNb‐MCM‐41 can retain its high catalytic activity over at least ten cycles of reactions without decrease of conversion of 2‐MTHF or selectivity to pentadienes. Additionally, the mechanistic aspects of activation and conversion of 2‐MTHF over AlNb‐MCM‐41 have been elucidated by employing operando X‐ray absorption spectroscopy (XAS), coupled with diffuse reflectance infrared Fourier transform spectroscopy (DRIFTS), and in situ high‐field solid‐state nuclear magnetic resonance (ssNMR) spectroscopy. The binding of 2‐MTHF on Nb^V^ sites has been revealed by ^93^Nb ssNMR, which represents the first ssNMR study of Nb^V^ sites in catalysis.

## Results and Discussion

### Synthesis and characterisation

**Figure 1:**
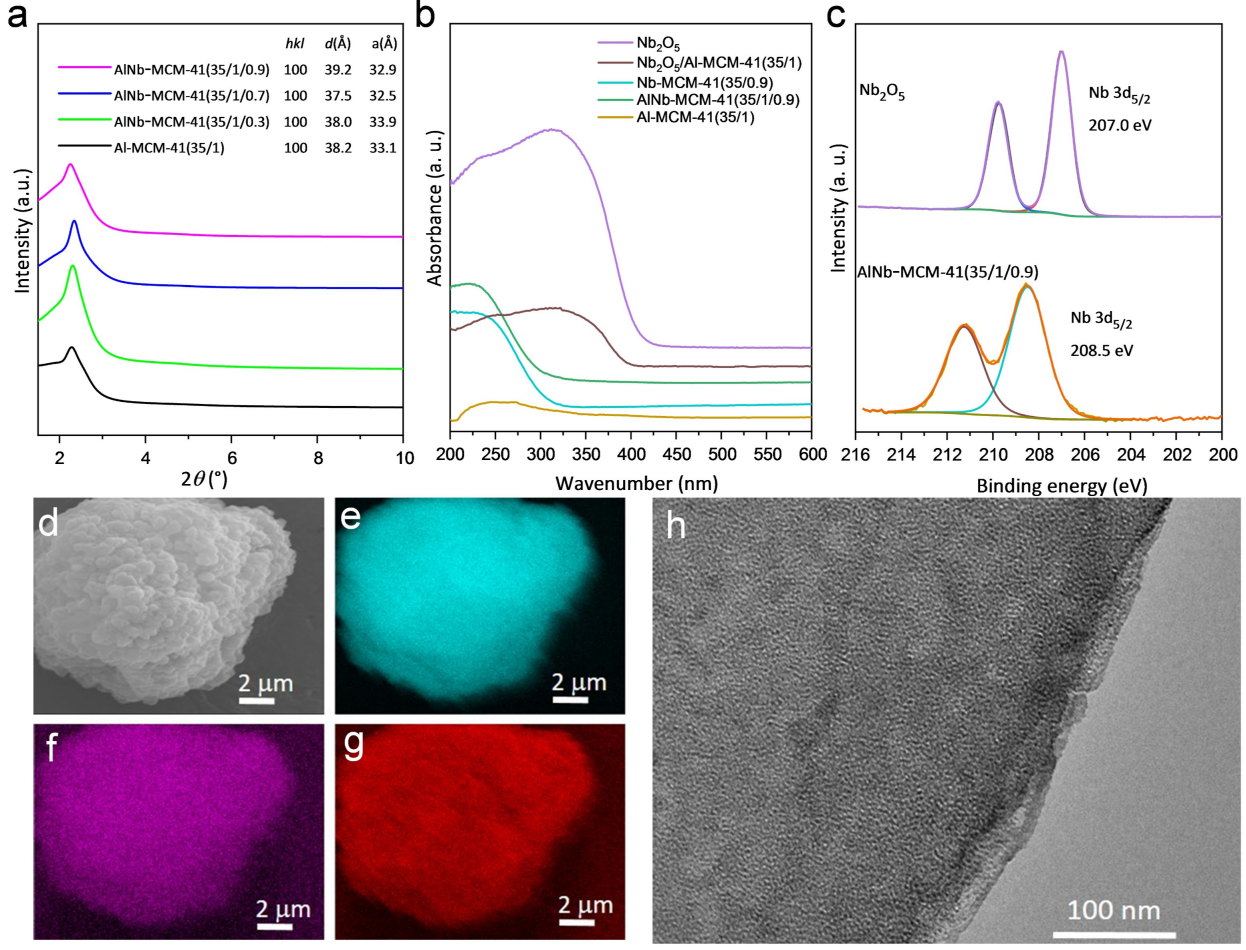
Physical characterization of catalysts. a) Comparison of small angle X‐ray scattering patterns of AlNb‐MCM‐41 and Al‐MCM‐41 (λ=1.54184 Å). b) UV‐vis spectra of Nb_2_O_5_, powered mixture of Nb_2_O_5_/Al‐MCM‐41(35/1), Nb‐MCM‐41(35/0.9), AlNb‐MCM‐41(35/1/0.9) and Al‐MCM‐41(35/1) samples. c) Nb 3d XPS spectra of Nb_2_O_5_ and AlNb‐MCM‐41 (35/1/0.9). d–g) EDX maps of Si Kɑ (e), Al Kɑ (f) and Nb Lɑ (g) in AlNb‐MCM‐41(35/1/0.9). h) TEM image of AlNb‐MCM‐41 (35/1/0.9).

AlNb‐MCM‐41 samples containing Nb^V^ and Al^III^ sites of varying Nb^V^ ratios (Si/Al/Nb=35/1/*x*, *x*=0.3, 0.7 and 0.9) have been synthesised from hydrothermal reactions at 100 °C for 3 days by using hexadecyltrimethyl‐ammonium bromide as the template. To the best of our knowledge, this represents the first example of (Nb, Al)‐bimetallic mesoporous silica where both Al^III^ and Nb^V^ sites locate at tetrahedral TO_4_ sites. For comparison, Al^III^‐free and Nb^V^‐free materials (Nb‐MCM‐41 and Al‐MCM‐41, respectively) have also been synthesised. All obtained materials exhibit an ordered two‐dimensional (2D) hexagonal framework structure (*a*=3.25–3.39 nm), as confirmed by small‐angle X‐ray diffraction (Figure 1a), and no Bragg peaks of Nb_2_O_5_ were observed (Figure S2). N_2_ adsorption isotherms at 77 K give Brunauer–Emmett–Teller (BET) surface areas of 1140–1346 m^2^ g^−1^ and pore‐wall thickness of 1.33–1.40 nm (Figure S3, Table S1). The apparent absorption band at 220 nm in the UV/Vis spectrum of AlNb‐MCM‐41(35/1/0.9) indicates the incorporation of Nb^V^ centres as tetrahedral [NbO_4_] moieties in the framework (Figure 1b).^[20]^ X‐ray photoelectron spectroscopy (XPS) confirms the binding energy of Nb^V^ 3d_5/2_ at 208.5 eV, which is higher than that (207.0 eV) of Nb_2_O_5_ (Figure 1c), indicating the strong electronic interaction between Nb^V^ sites and framework matrix.^[21]^ The presence of Si−O, Al−O and Nb−O species within AlNb‐MCM‐41(35/1/0.9) has been confirmed by O1s deconvoluted spectrum (Figure S5).^[22]^ Energy dispersive X‐ray (EDX) analysis identified the ratios of Si/Al/Nb and confirmed the homogeneous dispersion of Al^III^ and Nb^V^ sites within AlNb‐MCM‐41(35/1/0.9) (Figure 1d–g). Transmission electron microscopy (TEM) images of these samples confirmed the regular mesoporous structure and parallel channels, consistent with the structure of MCM‐41[^19^, ^29^] (Figure 1h, Figure S4a‐c). The environment of Al^III^ in AlNb‐MCM‐41 has been studied by high‐field (20.0 T) and fast magic angle spinning (MAS) ^27^Al ssNMR spectroscopy, which shows two major peaks (centred at delta{^27^Al} ≈55 and −2 ppm) that correspond to tetrahedral and octahedral coordination (respectively). This is again consistent with the parent Al‐MCM‐41 material (Figure S6). The ^1^H MAS ssNMR spectra show that the proton (including Brønsted acid) sites are very similar between Al‐MCM‐41 and AlNb‐MCM‐41(35/1/0.9) (Figure S6). Taken together, this suggests that any difference in the catalytic properties with the Nb‐doped samples must be mainly associated with the Nb^V^ site itself.

**Figure 2:**
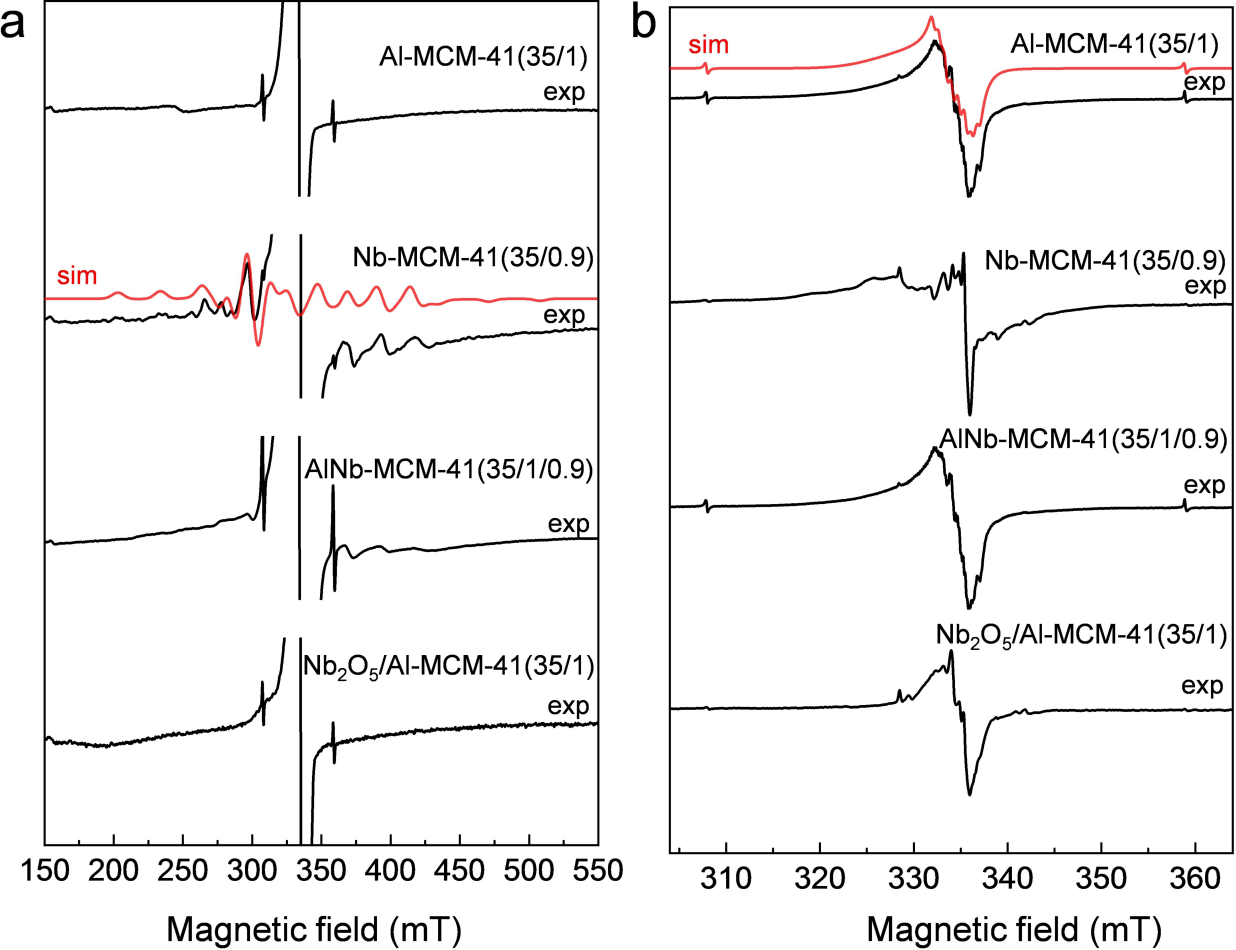
EPR spectra of Al‐MCM‐41(35/1), Nb‐MCM‐41(35/0.9), AlNb‐MCM‐41(35/1/0.9), and Nb_2_O_5_/Al‐MCM‐41(35/1). a) wide‐field and b) narrow‐field sweep spectra of the molecular sieves after γ‐irradiation; spectra measured at X‐band (ca. 9 GHz) and at 40 K. Simulated spectra of Nb^IV^ and Al−O⋅‐Si defects are presented as red dotted lines. The sharp, weak doublet appearing at ca. 310 and 360 mT is due to radiation‐induced trapped H atoms.

Electron paramagnetic resonance (EPR) spectroscopy was used to prove the successful incorporation of Nb^V^ and Al^III^ into framework sites. Although Nb‐MCM‐41 (the isostructural analogue with 0 % Al loading) and AlNb‐MCM‐41 are EPR silent due to the diamagnetic Nb^V^ and Al^III^ ions, paramagnetic centres characteristic of tetrahedral [Nb^IV^O_4_] units and oxygen electron‐hole defects neighbouring Al nuclei can be induced by γ‐irradiation of the samples.^[23]^ γ‐irradiation induces electronic defects, rather than gross structural changes, and thus such observations give structural information on the parent material. X‐band EPR spectra were collected at 40 K on paramagnetic defect‐containing γ‐irradiated metal‐dope mesoporous silicas. The spectrum of Nb‐MCM‐41 (35/0.9) (Figure 2a) reveals a characteristic signal for Nb^IV^ (observed over a wider field sweep) with a 10‐line hyperfine structure (^93^Nb, I=9/2, 100 % natural abundance).[^17^, ^23^] The Nb^IV^ signal can be simulated assuming axial site symmetry with electronic *g*‐values and ^93^Nb hyperfine interaction (A^Nb^) *g*⊥=1.936, *g*||=1.877 and A^Nb^⊥=159 G (0.0148 cm^−1^), A^Nb^||=318 G (0.0297 cm^−1^). These values are in close agreement with those reported for Nb^IV^ centres generated on pseudo‐tetrahedral NbO_4_ units in the framework of NbAlS‐1 and silicalite‐1 by γ‐irradiation.[^13^, ^23^] This Nb^IV^ signal is also observed in AlNb‐MCM‐41(35/1/0.9), but not in Al‐MCM‐41(35/1) nor a powered mixture of Nb_2_O_5_ with Al‐MCM‐41(35/1), giving strong evidence for incorporation of niobium into the framework sites of MCM‐41 (Figure 2a). All the spectra also show a much narrower signal due to radiation‐induced electron hole defects (Figure 2b). For Al‐MCM‐41(35/1), this signal shows hyperfine structure due to ^27^Al (I=5/2, 100 % natural abundance) and is characteristic of an Al−O⋅−Si site,^[24]^ with *g* and hyperfine values close to those previously reported (*g*=2.003, 2.012, 2.045; A^Al^=7, 7 G, third component unresolved). Observation of the same signal in AlNb‐MCM‐41, and the absence of the ^27^Al structure on the equivalent signal in Nb‐MCM‐41(35/0.9) (which only has Si−O−Si sites) proves that Al^III^ is also incorporated into framework sites of the MCM‐41 structure (Figure 2b). Thus, EPR measurements provide convincing evidence for the incorporation of both Nb^V^ and Al^III^ sites into the MCM‐41 framework. Similar results are obtained for materials with lower Nb^V^ content AlNb‐MCM‐41(35/1/0.7) and AlNb‐MCM‐41(35/1/0.3) (Figure S7).

The acidity of these catalysts has been quantified by temperature‐programmed desorption of ammonia (NH_3_‐TPD) (Figure S8 and Table S2). Al‐MCM‐41(35/1) shows both weak and strong acid sites with the loading of 0.147 and 0.063 mmol g^−1^, respectively, whereas Nb‐MCM‐41(35/0.9), in absence of Al^III^ sites, exhibits only weak acid sites of 0.045 mmol g^−1^. For the powdered mixture of Nb_2_O_5_/Al‐MCM‐41(35/1) with the Si/Al/Nb ratio of 35/1/0.9, the concentrations of both strong and weak acid sites are consistent with that of Al‐MCM‐41(35/1). Interestingly, by incorporating both Nb^V^ and Al^III^ sites, AlNb‐MCM‐41 (35/1/*x*) (*x*=0.3, 0.7, 0.9) shows higher concentrations of weak acid sites upon increased loading of Nb^V^ (0.186, 0.231 and 0.258 mmol g^−1^ for *x*=0.3, 0.7 and 0.9, respectively) and a lower concentration of strong acid sites (0.024 mmol g^−1^) compared with that of Al‐MCM‐41 (35/1). The nature of these acid sites has been determined by d_3_‐acetonitrile DRIFTS experiments, which confirmed that the concentration of Brønsted acid sites increases upon the introduction of Nb^V^ into Al‐MCM‐41(35/1)[^25^, ^26^] (Figure S9). AlNb‐MCM‐41(35/1/0.9) shows only weak Lewis acidity, which is distinct to Al‐MCM‐41 that displays strong Lewis acidity. The oxygen affinity of AlNb‐MCM‐41 was verified by acetone adsorption‐desorption DRIFTS experiments^[13]^ (Figure S10). The stretching vibration of the C=O group in acetone is at 1740 cm^−1^. Upon adsorption followed by desorption by purging N_2_ for 30 minutes on AlNb‐MCM‐41(35/1/0.9), AlNb‐MCM‐41(35/1/0.7), AlNb‐MCM‐41(35/1/0.3), Nb‐MCM‐41(35/0.9) and Al‐MCM‐41(35/1), this band is red‐shifted to 1705, 1706, 1706, 1706 and 1708 cm^−1^, respectively, indicating that Nb^V^ sites show enhanced activation ability of the C=O bond upon adsorption. Upon purging by N_2_ at 150 °C for a further 10 minutes, all acetone molecules are desorbed from Al‐MCM‐41(35/1) completely, whereas on Nb‐containing MCM‐41 materials, the C=O stretching band is retained, again confirming the strong binding of acetone molecules to the Nb^V^ sites. Thus, the introduction of Nb^V^ sites in MCM‐41 can afford significantly strong affinity for oxygen‐containing molecules.

### Catalytic tests

**Figure 3:**
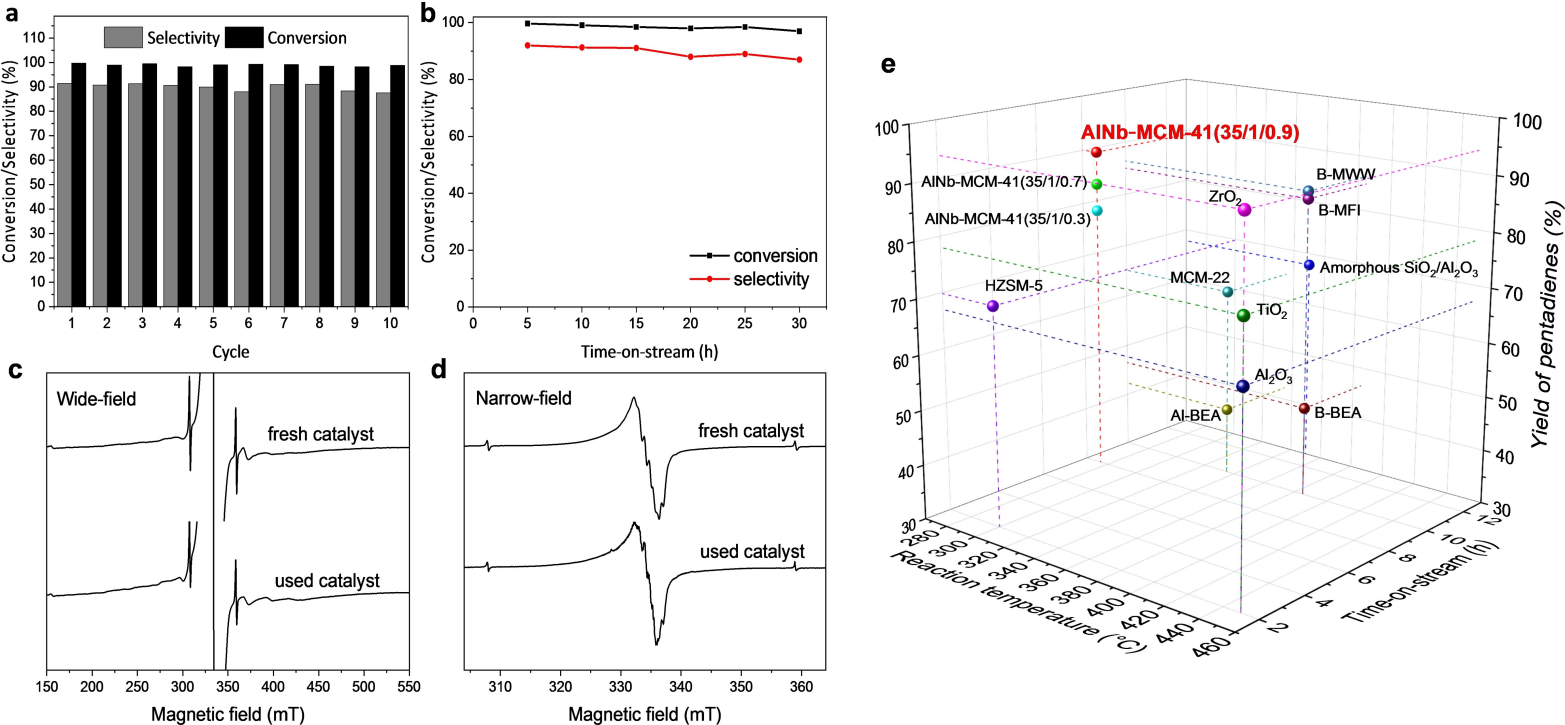
Catalysts stability. a) Comparison of the selectivity to pentadienes over 10 cycles of reactions over AlNb‐MCM‐41(35/1/0.9), Reaction conditions: catalyst, 0.5 g; reaction temperature, 275 °C; atmospheric pressure; Weight Hourly Space Velocity (WHSV), 0.12 h^−1^; time‐on‐stream=8 h. b) Lifetime study of AlNb‐MCM‐41(35/1/0.9) at 275 °C. c) Wide‐field and d) narrow‐field sweep X‐band EPR spectra (measured at 40 K) of the fresh and used AlNb‐MCM‐41(35/1/0.9) after γ‐irradiation. e) Comparison of the catalytic performance of reported catalysts for the conversion of 2‐MTHF to pentadienes (full data is listed in Table S4).[^5^, ^9^, ^28^]

The conversion of 2‐MTHF to pentadienes was conducted using a fixed‐bed reactor packed with these catalysts under continuous flow conditions at 275 °C (Table 1). Nb‐MCM‐41(35/0.9) (entry 5) shows a low conversion of 2‐MTHF at 73 % and a poor selectivity to pentadienes of 22 % due to the low concentration of acid sites (0.045 mmol g^−1^) and lack of Brønsted acid sites (Figure S9a, Table S2). Meanwhile, considerable amounts of by‐products (up to 72 %), such as pentenols, pentanal and pentenals, were observed owing to the promoted activation of C−O bonds on the Nb^V^ sites. Al‐MCM‐41(35/1) shows high conversion of 2‐MTHF at 98 % and increased selectivity to pentadienes of 61 % (entry 4) as a result of the increased concentration of acid sites (0.21 mmol g^−1^). The bimetallic AlNb‐MCM‐41 materials all show quantitative conversion (>99 %) of 2‐MTHF and high selectivities to pentadienes (80–91 %) (entries 1–3). The selectivity to pentadienes increases with the increase of loading of Nb^V^ sites. The selectivities to 1, 3‐pentadiene (*cis* and *trans* combined) and 1, 4‐pentadiene reached 75 % and 16 %, respectively, over AlNb‐MCM‐41(35/1/0.9) at full conversion (Figure S11). Importantly, the catalytic performance of AlNb‐MCM‐41(35/1/0.9) compares favourably with all heterogeneous catalysts reported for this reaction to date (Figure 3e, Table S4). Although NbOPO_4_ shows quantitative conversion of 2‐MTHF and high selectivity to pentadienes (80 %) (entry 8), it exhibits a much lower utilisation efficiency of Nb^V^ compared with AlNb‐MCM‐41(35/1/0.9); the concentration of Nb^V^ within these catalysts is 46 % and 3.7 %, respectively. Thus, AlNb‐MCM‐41 with atomically‐dispersed Nb^V^ sites can maximise the utilisation of Nb^V^ sites and catalyst stability. The excellent stability of AlNb‐MCM‐41(35/1/0.9) has been demonstrated by both cycling and time‐on‐stream tests. After each cycle, the catalyst is calcined at 550 °C in a flow of air for 14 hours. No apparent decrease in the conversion of 2‐MTHF or selectivity to pentadienes was observed over 10 cycles of the reaction (Figure 3a). A lifetime study of AlNb‐MCM‐41(35/1/0.9) has been conducted for 30 hours, and stable production of pentadienes is observed (Figure 3b). The retention of Nb^V^ and Al^III^ sites within the framework of AlNb‐MCM‐41(35/1/0.9) catalyst used after ten cycles of the reaction was confirmed by EPR spectroscopy after γ‐irradiation of the recycled catalyst (Figure 3c–d).

**Table 1:** Summary of the conversion of 2‐MTHF and selectivity of products over different catalysts.^[a]^

Entry	Catalyst	Conversion [%]	Pentadienes	Butenes	Propylene	Others^[b]^
			Carbon selectivity [%]			
1	AlNb‐MCM‐41(35/1/0.9)	99	91	6	1	2
2	AlNb‐MCM‐41(35/1/0.7)	99	85	9	3	3
3	AlNb‐MCM‐41(35/1/0.3)	99	80	10	3	7
4	Al‐MCM‐41(35/1)	98	61	10	2	27
5	Nb‐MCM‐41(35/0.9)	73	22	5	1	72
6	Nb_2_O_5_/Al‐MCM‐41(35/1)^c^	97	66	9	2	23
7	Nb_2_O_5_	86	27	2	1	70
8	NbOPO_4_	99	80	6	4	10

[a] Reaction conditions: catalyst, 0.5 g; reaction temperature, 275 °C; atmospheric pressure; Weight Hourly Space Velocity (WHSV), 0.12 h^−1^; time‐on‐stream=8 h. [b] Other products include pentenols, pentanal, pentenals and higher hydrocarbons. ^c^The ratio of Si/Al/Nb in the powdered mixture of Nb_2_O_5_/Al‐MCM‐41(35/1) (5.3 wt %/94.7 wt %) is 35/1/0.9.

### Studies of the reaction mechanism

**Figure 4:**
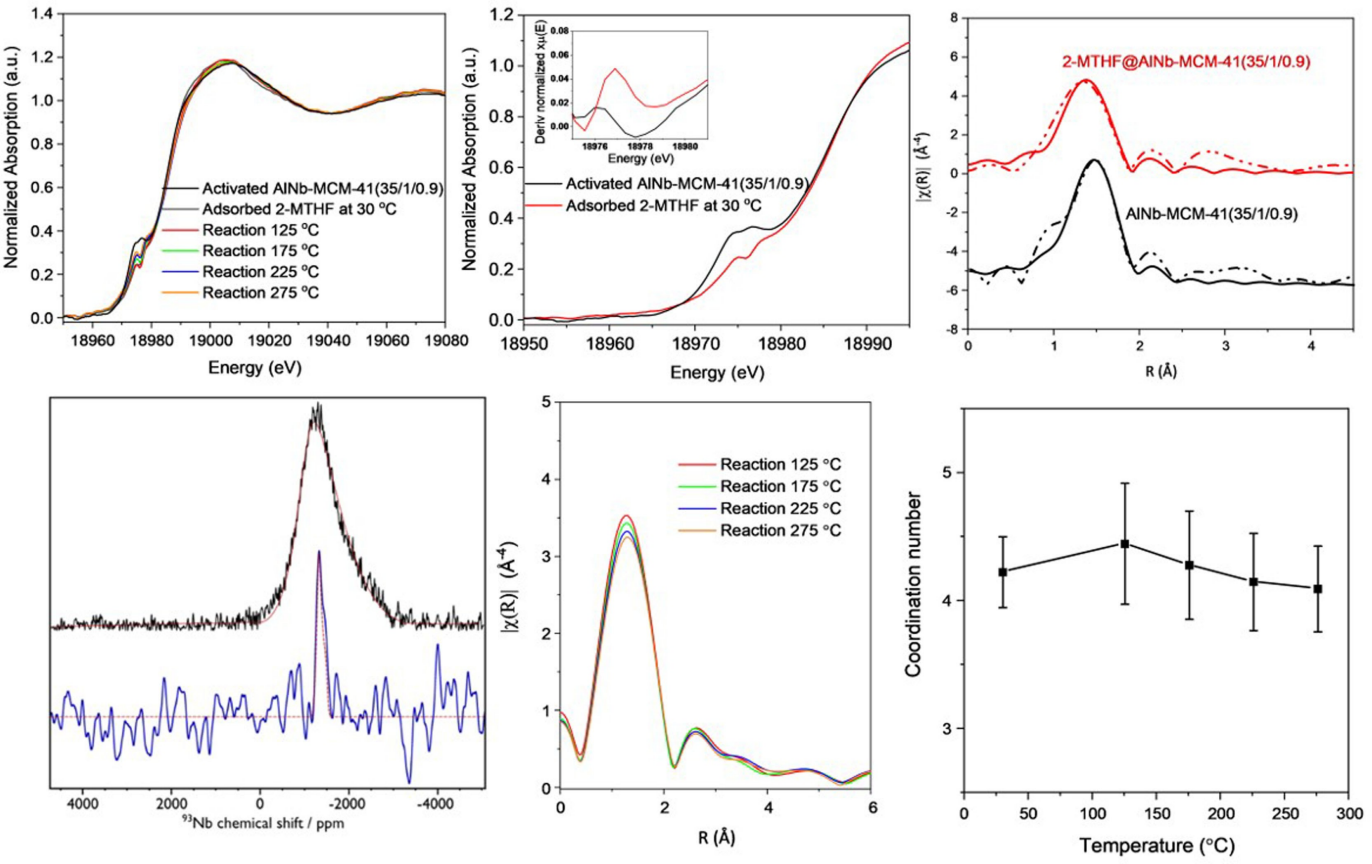
a) Normalised Nb K‐edge XANES spectra of fresh and 2‐MTHF‐adsorbed AlNb‐MCM‐41(35/1/0.9) reacted at different temperatures. b) Shifts of the pre‐edge feature in AlNb‐MCM‐41(35/1/0.9), with inset showing the first derivate in the presence of 2‐MTHF. c) Non‐phase corrected Fourier transformed Nb K‐edge EXAFS spectra (dotted lines) and fitting curves (solid lines) for activated and 2‐MTHF adsorbed AlNb‐MCM‐41(35/1/0.9) in R spaces of 1.0‐3.0 Å for AlNb‐MCM‐41 and 1.0‐2.5 Å for 2MTHF adsorbed AlNb‐MCM‐41, respectively . d) ^93^Nb static solid‐echo NMR spectra of pristine AlNb‐MCM‐41(35/1/0.9) (black) and the same material with adsorbed 2‐MTHF (blue). Corresponding simulated spectra (red) use the following (non‐zero) parameters: *C*_Q_=30 MHz, *h*_Q_=1, *d*_iso_=−1250 ppm (2‐MTHF@AlNb‐MCM‐41(35/1/0.9)) and *C*_Q,max._=93 MHz, *d*_iso_=−1172 ppm with a Czjzek Gaussian Isotropic Model used for the distribution (AlNb‐MCM‐41(35/1/0.9)). e) Non‐phase corrected Fourier transformed Nb K‐edge EXAFS spectra for the reaction with adsorbed 2‐MTHF on AlNb‐MCM‐41(35/1/0.9) at different temperatures. f) Coordination number of Nb−O bonds (obtained by EXAFS fitting of the first shell data) during the reaction at different temperature.

**Figure 5:**
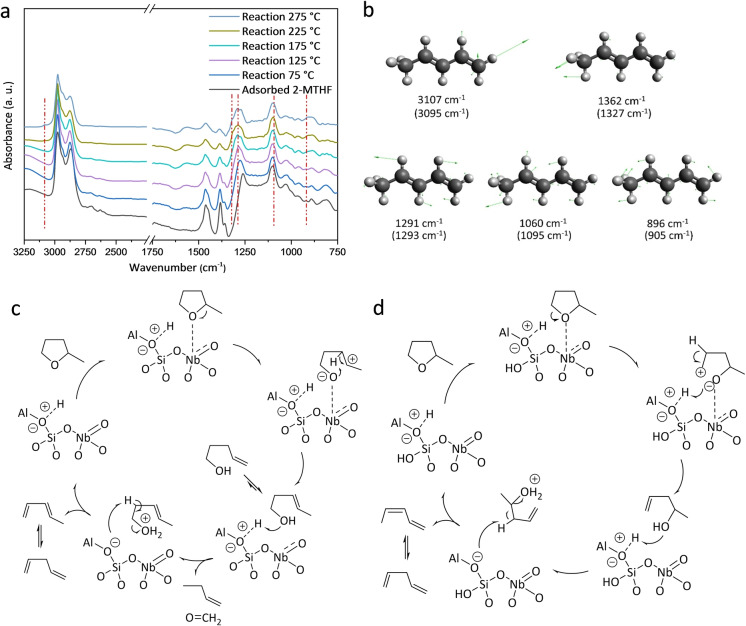
Operando DRIFTS spectra of AlNb‐MCM‐41(35/1/0.9) on the adsorption and catalytic conversion of 2‐MTHF and proposed reaction mechanism. a) Comparison of DRIFTS spectra for adsorbed and reacted 2‐MTHF on AlNb‐MCM‐41(35/1/0.9) at different temperatures. b) Selected vibrational modes of 2‐MTHF. The wavenumbers of adsorbed 2‐MTHF as observed by experiments are shown in parentheses. Proposed reaction mechanisms for conversion of 2‐MTHF over AlNb‐MCM‐41c) Primary pathway and d) Secondary pathway, based on the analysis of the experimental results and literature.[^5^, ^9^, ^27^]

Energy dispersive X‐ray absorption spectroscopy (EDXAS) at the Nb K‐edge, coupled with DRIFTS measurements, was measured to study the effect of active Nb^V^ sites in the adsorption and conversion of 2‐MTHF over AlNb‐MCM‐41 (35/1/0.9) (Figures 4–5). X‐ray absorption near‐edge spectra (XANES) of AlNb‐MCM‐41(35/1/0.9) indicate the pentavalent oxidation state of Nb^V^ centres in all samples (Figure 4a).^[31]^ The presence of an apparent pre‐edge peak in the spectrum of the activated AlNb‐MCM‐41(35/1/0.9) sample is attributed to the dipole allowed transition from the 1*s* to *t*2 level that is present in tetra‐coordinated Nb^V^ centers.[^30^, ^32^] On adsorption of 2‐MTHF, the pre‐edge of Nb is blue‐shifted by ≈0.7 eV due to the electron donated from 2‐MTHF, reflecting a direct interaction between the Nb^V^ sites and the 2‐MTHF substrate^[17]^ (Figure 4b insert). Furthermore, the reduced pre‐edge intensity in the XANES spectrum of the 2‐MTHF adsorbed sample suggests a more symmetrical geometry of the Nb^V^ center. This is verified through the fitting of the first coordination shell of the extended X‐ray absorption fine structure (EXAFS). Fourier transformed (FT) EXAFS fitting results show that the difference in distances of Nb=O and Nb−O bonds decreases in AlNb‐MCM‐41(35/1/0.9) upon binding of 2‐MTHF. This suggests a substantial decrease in the double‐bond nature of Nb=O groups upon the adsorption of 2‐MTHF,^[31]^ consistent with the decrease of pre‐edge peaks in XANES (Figure4c, Table S5). The finding is further validated through high‐field (20.0 T) ^93^Nb ssNMR spectroscopy (Figure 4d), which shows a broad signal consistent with non‐centrosymmetric tetra‐coordinated (i.e., NbO_4_) Nb^V^ environments^[33]^ for pristine AlNb‐MCM‐41(35/1/0.9) but a much narrower signal consistent with penta‐coordinated (i.e., NbO_5_) Nb^V[33]^ upon adsorption of 2‐MTHF. Moreover, this high‐field ssNMR study shows that there is no change in the ^27^Al environment upon adsorption of 2‐MTHF but that the narrow ^1^H signals from BASs (Brønsted acid sites) disappear (Figure S6). This indicates that Al^III^ sites play little role during the initial stages of adsorption and reaction and that BASs are involved.

The reaction of 2‐MTHF (from 30 to 275 °C) has also been examined by operando EXAFS analysis (Figure 4e–f, Figure S13). Upon the increase of temperature, the coordination number of Nb^V^ sites increases slightly at 125 °C under the constant 2‐MTHF feeding, indicating strong adsorption of 2‐MTHF on Nb^V^ sites. It then decreases due to the conversion of 2‐MTHF to dienes that can desorb readily to regenerate the 4‐coordianted Nb^V^ sites. This is also observed in the analysis of exhaust gas composition by online mass spectrometry (Figure S14).

The EDXAS‐coupled DRIFTS study (Figure 5a) shows that with the increase of temperature and reaction time, the small shoulders at 1385 and 1450 cm^−1^, assigned to C−H bending vibration of the methyl group and −CH_2_‐ scissoring vibration of the ring of 2‐MTHF, decrease in intensity. Additionally, the bands at 2962, 2933 and 2875 cm^−1^ (assigned to stretching modes of −CH‐, −CH_2_‐, −CH_3_ groups of adsorbed alcohols, alkanes and reactant)^[27]^ also decrease in intensity. When the temperature is increased to 175 °C, new peaks appear at 3095, 1327, 1293, 1095 and 896 cm^−1^ (Figure 5b), corresponding to the symmetrical =C−H stretching, methyl rock, −CH= bending, C−CH_3_ stretching, =CH_2_ wagging of *trans*‐1,3 pentadienes, respectively, consistent with the result of mass spectroscopy (Figure S14). The conversion of 2‐MTHF over NbAl‐MCM‐41(35/1/0.9) was also conducted in a batch reactor as a function of reaction time to identify the intermediates (Figure S15). The catalytic network has been established based upon the analysis of structure, adsorption and literature (Figure 5c and 5d).[^5^, ^9^, ^27^, ^28^] Over AlNb‐MCM‐41, the adsorption of 2‐MTHF occurs on Nb^V^ sites via the O‐centre, and this is followed by the ring‐opening of 2‐MTHF at elevated temperatures through the cleavage of C−O bonds. This results in two different reaction pathways, yielding a secondary carbenium ion (Figure 5c) or a primary carbenium ion (Figure 5d) with a hydroxyl group. According to the carbenium character of the kinetically relevant transition state, the ring‐opening process to generate secondary carbenium is more favourable.^[9]^ This is further confirmed in the batch‐mode catalytic tests as a function of time (Figure S15), where 3‐pentenol is the main intermediate during the reaction. For the primary reaction pathway, the 3‐pentenol and 4‐pentenol formed upon ring‐opening of 2‐MTHF can undergo dehydration to form pentadienes. For the secondary pathway, concerted hydride shift occurs, which is followed by a dehydration step to pentadienes driven by the Brønsted acid.

## Conclusion

The development of new mesoporous catalysts with tunable acid sites is an important but challenging task to drive the efficient utilisation of biomass and derived materials on the roadmap to carbon neutrality. We report the synthesis of a series of new (Al,Nb)‐bimetallic mesoporous silica materials for the first time. The simultaneous incorporation and tuning of the ratio of Nb^V^ and Al^III^ sites results in an optimal nature and distribution of acid sites within AlNb‐MCM‐41 compared with the conventional Al‐MCM‐41 structure. AlNb‐MCM‐41(35/1/0.9) shows excellent catalytic performance for the conversion of biomass‐derived 2‐MTHF to pentadienes with a high selectivity of 91 % at full conversion at 275 °C and 1 atm. The important role of Nb^V^ sites and optimal weak acidity of AlNb‐MCM‐41 have been confirmed by a combination of operando and in situ experiments, which highlight the positive effect of Nb^V^ sites on binding and activation of 2‐MTHF in the production of pentadienes.

## Conflict of interest

The authors declare no conflict of interest.

1

## Supporting information

As a service to our authors and readers, this journal provides supporting information supplied by the authors. Such materials are peer reviewed and may be re‐organized for online delivery, but are not copy‐edited or typeset. Technical support issues arising from supporting information (other than missing files) should be addressed to the authors.

Supporting Information

## Data Availability

The data that support the findings of this study are available in the supplementary material of this article.
